# Rapid Diagnostic Tests for Neglected Infectious Diseases: Case Study Highlights Need for Customer Awareness and Postmarket Surveillance

**DOI:** 10.1371/journal.pntd.0004655

**Published:** 2016-11-03

**Authors:** Barbara Barbé, Kristien Verdonck, Sayda El-Safi, Basudha Khanal, Syna Teav, Jean-Roger Lilo Kalo, Raffaella Ravinetto, François Chappuis, Marleen Boelaert, Jan Jacobs

**Affiliations:** 1 Institute of Tropical Medicine, Antwerp, Belgium; 2 Faculty of Medicine, University of Khartoum, Khartoum, Sudan; 3 B.P. Koirala Institute of Health Science, Dharan, Nepal; 4 Department of Infectious Diseases, Sihanouk Hospital Center of HOPE, Phnom Penh, Cambodia; 5 Institut National de Recherche Biomédicale, Kinshasa, Democratic Republic of the Congo; 6 Clinical Pharmacology and Pharmacotherapy, KU Leuven, Leuven, Belgium; 7 Geneva University Hospitals, Geneva, Switzerland; 8 Department of Microbiology and Immunology, KU Leuven, Leuven, Belgium; Foundation for Innovative New Diagnostics (FIND), SWITZERLAND

## Introduction

Accurate diagnosis of infectious diseases is essential for appropriate targeting of treatment and disease control. Rapid diagnostic tests (RDTs) are quick and easy to perform, they give results during one clinic visit, and they can be used in settings with little infrastructure or trained personnel. RDTs are promising tools to improve diagnosis in remote or low-resource settings. In the field of neglected infectious diseases, new manufacturers, RDTs, and users are coming onto the scene [1, 2]. As access to RDTs improves, the need for quality assurance and postmarket surveillance increases.

The International Medical Device Regulators Forum has formulated guidelines about quality assurance of medical devices, including RDTs, which have been adopted as regulatory standards in Australia, Canada, the European Union, Japan, and the United States [3]. Specific quality standards for in vitro diagnostic tests (IVDs) (ISO 13485:2003) and medical laboratories (ISO 15189:2012) have been published by the International Organisation for Standardisation [4, 5]. In less-regulated settings, the World Health Organisation (WHO) has stepped in to promote IVD quality [3, 6, 7].

Participation from various stakeholders is required to assure RDT quality. Manufacturers must ensure that their products are ready for the market—i.e., that product design, development, testing, manufacturing, packaging, and labelling meet the required standards of safety and performance. The role of RDT users is, among other responsibilities, to know indications, contraindications, and operating procedures of the devices. Most regulatory authorities recognise that efficient communication between manufacturers and users is key to postmarket surveillance [3–7]. In low-resource settings and in the field of neglected infectious diseases, this communication between manufacturers and users may be suboptimal, as well as the pre- and postmarketing oversight of national regulatory authorities.

The Neglected Infectious Diseases dIAGnosis (NIDIAG) consortium aims to improve diagnostic approaches for different clinical syndromes in low-resource settings where neglected infectious diseases are prevalent. In this case study, we assessed several quality aspects of RDTs used in the NIDIAG study about persistent fever: we focused on RDT labelling and instructions for use (IFU) and on product-related incidents, including communication with manufacturers about these incidents.

## Methods

The NIDIAG study about persistent (≥7 days) fever recruited 1,926 patients in four countries (Sudan, the Democratic Republic of the Congo, Nepal, and Cambodia). The study evaluated 11 RDTs from seven manufacturers targeting human African trypanosomiasis, leptospirosis, malaria, typhoid fever, and visceral leishmaniasis (Table 1). These RDTs were selected by an expert committee based on their potential to improve prognosis of patients with persistent fever in the study areas. To be included as index tests, the RDTs had to target severe and treatable infectious diseases that cause persistent fever and they had to be ready for evaluation in phase III or phase IV diagnostic studies.

**Table 1:** Characteristics of 11 rapid diagnostic tests used in the NIDIAG persistent fever study.

Target condition	Product name (manufacturer, country of headquarters)	Product code	Test format	Target type	CE mark^a^
Human African trypanosomiasis	HAT Sero K-SeT (Coris BioConcept, Belgium)	K-12S2	Cassette	Antibody	Yes
Human African trypanosomiasis	SD Bioline HAT (Standard Diagnostics, Inc., Republic of Korea)	53FK10	Cassette	Antibody (IgG/IgM/IgA)	Yes
Leptospirosis	Test-it^TM^ Leptospira IgM Lateral Flow Assay (Life Assay Diagnostics [Pty] Ltd, South Africa)	LEP001	Cassette	Antibody (IgM)	No
Leptospirosis	SD Bioline Leptospira IgG/IgM (Standard Diagnostics, Republic of Korea)	16FK40	Cassette	Antibody (IgG/IgM)	Yes
Malaria	SD Bioline Malaria Antigen P.f/Pan (Standard Diagnostics, South Africa)	05FK60	Cassette	Antigen (Pf-HRP2, pan-pLDH)	Yes
Malaria	CareStart^TM^ Malaria pLDH 3 lines (pan/Pf) (Access Bio, Inc., US)	G0121	Cassette	Antigen(Pf-pLDH, pan-pLDH)	Yes
Typhoid fever	Typhidot Rapid IgM (Reszon Diagnostics International, Malaysia)	RTF-RD010	Cassette	Antibody (IgM)	No
Typhoid fever	Test-it^TM^ Typhoid IgM Lateral Flow Assay (Life Assay Diagnostics [Pty] Ltd, South Africa)	TYP001	Cassette	Antibody (IgM)	No
Typhoid fever	SD Bioline Salmonella typhi IgG/IgM (Standard Diagnostics, Republic of Korea)	15FK12	Dipstick^b^	Antibody (IgG/IgM)	No
Visceral leishmaniasis	Dynamic Flow Visceral Leishmania IgG Antibody Card Test (Ease Medtrend Biotech, Ltd., China)	1-126G-DF-W	Cassette	Antibody (IgG)	No
Visceral leishmaniasis	IT LEISH (Bio-Rad, US)	710124	Hybrid^c^	Antibody	Yes

Ig, immunoglobulin; Pf-HRP2, *Plasmodium falciparum* histidine-rich protein-2; pan-pLDH, pan *Plasmodium-*specific parasite lactate dehydrogenase; Pf-pLDH, *Plasmodium falciparum*-specific parasite lactate dehydrogenase

^a^The CE mark indicates that the manufacturer or importer claims compliance with European legislation.

^b^Rapid diagnostic test device consisting of only the nitrocellulose strip that is placed on or in a platform (tube, backing paper, etc.).

^c^Test device consisting of a nitrocellulose strip that is dipped into a plastic housing with buffer and specimen wells.

The assessment of RDT labelling and IFU was done by one trained person using a checklist that had been developed previously based on international regulatory documents, a review of the literature, field observations, and comments from manufacturers and implementers. This checklist is given as supporting information (S1 Table) [8]. The product-related incidents were reported by the laboratory technicians in the study sites. They had received on-the-job training to correctly manipulate and interpret the RDTs and to follow standard operating procedures in line with good clinical laboratory practice guidelines. The diagnostic accuracy (sensitivity and specificity) of the RDTs is not included in this report.

The following definitions were used. Test results were invalid if there was no control line. Migration was considered to be delayed if the sample/buffer mixture did not start to migrate immediately (this usually takes only a few seconds). Poor background clearing was defined as the presence of a red-coloured background in the result window of the test strip at the time of result interpretation.

## Results

**Fig 1:**
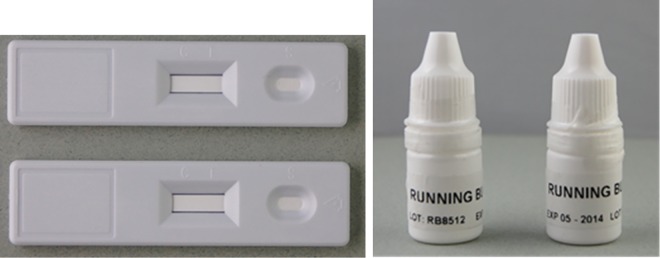
Cassettes and buffer bottles from different products but indistinguishable from each other.

The results for adherence to recommended RDT labelling and IFU are shown in Table 2. Five of the 11 RDT products had a meaningful name (i.e., mentioning at least the target condition and the type of test). Ten products displayed internationally recognized symbols, but none of them provided a complete symbol key. Four products displayed all essential information on the box. The cassettes from three products and buffer bottles from five products were correctly labelled. Fig 1 shows cassettes and buffer bottles for leptospirosis and typhoid fever tests from the same manufacturer, which are indistinguishable from each other. Four RDT products included appropriately labelled lancets. The IFU of eight RDT products included all required information but were not sufficiently readable (font size too small, closed letter type, and/or too difficult phrasing precluding easy reading and understanding). In summary, none of the 11 RDTs complied with all the specifications.

**Table 2:** Assessment of labelling and instructions for use of 11 rapid diagnostic tests used in the NIDIAG persistent fever study.

Criteria^a^	Number of RDT products complying with the criteria	Comment
The product has a meaningful name (mentioning at least the target condition and the type of test)	Five products from three manufacturers	For none of the RDT products, the items included in the kit were consistently labelled (different product name on RDT box, device packaging, and buffer bottle).
If internationally recognized symbols are used, a complete symbol key^b^ is provided	Zero products from zero manufacturers	Internationally recognized symbols were displayed for ten products from six manufacturers. A symbol key was provided for four products from three manufacturers, but this was incomplete.
Labels and prints are durable (humidity-proof and well-fixed)	Eight products from five manufacturers	This criterion was not evaluated for one product because it did not display any labelling on the outer packaging.
The list of components is displayed on the box	Four products from three manufacturers	This criterion was not evaluated for one product because it did not display any labelling on the outer packaging.
Essential product information is printed on the box (product name, reference number, lot number, expiration date, storage temperature, number of tests included)	Six products from four manufacturers	This criterion was not evaluated for one product because it did not display any labelling on the outer packaging.One product displayed a different product name on the box than the one printed in the IFU. The remaining nine products displayed the same product name on the box as in the IFU.
The cassette is appropriately labelled (target condition and single printed reading legend) and has enough place to write patient identity information	Three products from two manufacturers	One RDT with dipstick format was not taken into account for this criterion.Seven products did not comply with the criterion:- five products from four manufacturers did not display the target condition on the cassette (three of which did not display any information);- one had two reading legends instead of one- one did not have enough place to write patient identity information.
The buffer bottle is appropriately labelled (product name, lot number, and expiration date)	Five products from two manufacturers	In addition to product name, lot number, and expiration date, four products from one manufacturer also displayed manufacturer name, storage conditions, and buffer volume (which is correct practice).
If included, transfer devices are of the appropriate type	Eight products from four manufacturers	Transfer devices were not included in two RDT kits.A glass capillary tube was included for one product, which is not recommended because of risk of cuts. One of the two malaria RDT products included an inverted cup, which is one of the recommended transfer devices for malaria RDTs [9]. The remaining RDT products included plastic pipettes and straws with an indication line (six products from four manufacturers) and loops (one product).For one product, the packaging displayed the volume of the transfer device and "single use."
If included, lancets are appropriately labelled (lot number, expiration date, and a symbol and/or word for sterile product and single use)	Four products from two manufacturers	Lancets were not included in five RDT kits.
If included, alcohol swabs are appropriately labelled (lot number and expiration date)	Two products from two manufacturers	Five RDT kits did not include alcohol swabs.The remaining six RDT kits included alcohol swabs, which were easy to open (without need for scissors), and mentioned on the packaging the type and concentration of the alcohol used. Two of these also mentioned lot number and expiration date.
IFU are complete (full contents of the kit, the test procedure, correct interpretation of the test results, version number, and date of issue)	Eight products from six manufacturers	Instructions for use were not provided in paper format but through email for one RDT product, which is not practical, certainly not in low-resource settings. None of the IFU displayed track changes or a revision history.
IFU for use are readable (font size ≥9, interline space ≥2, open letter type and Flesch–Kincaid grade^c^ <6)	Zero products from zero manufacturers	None of the IFU had a font size ≥9 or a Flesch–Kincaid grade <6. The IFU of seven products from three manufacturers had an open letter type. All IFU had an interline space ≥2.

^a^The criteria are based on a review of international regulatory documents and scientific literature, complemented with field observations and comments from manufacturers and implementers (S1 Table) [8].

^b^A symbol key is a glossary with explanations of the symbols.

^c^The Flesch–Kincaid grade expresses the reading competence needed to understand a given text.

**Fig 2:**
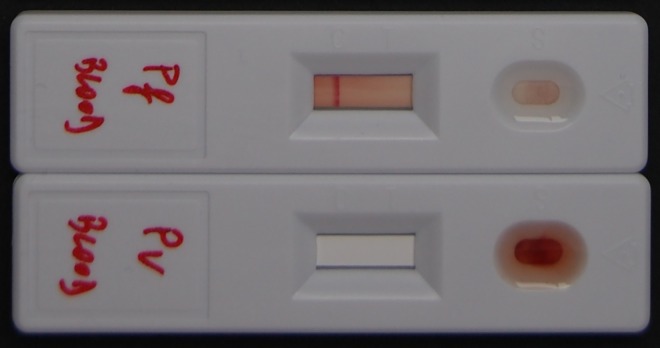
Above: Rapid diagnostic test with correct lateral flow and timely migration of sample/buffer mixture. Below: Rapid diagnostic test with blocked lateral flow and no or delayed migration. The sample/buffer mixture is not flowing through the test strip but is staying behind in the sample well. This is considered as an invalid result.

**Fig 3:**
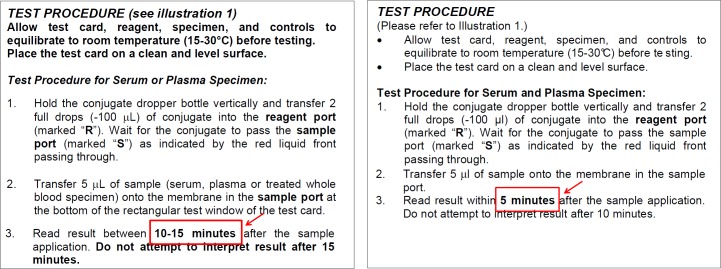
Two successive versions of the instructions for use of a rapid diagnostic test (most recent version on the right). The change in reading time (indicated with a red arrow on the picture) was not communicated to the user in writing, by an alert on the box, or through a new version number, “track changes,” or a revision history of the instructions for use. Only the date of issue of the instructions for use had changed.

We registered 15 quality-related incidents for five RDT products: one related to supply (insufficient buffer volume), three to shipment (lack of temperature monitoring or shipment at too high temperature), and one to storage (elevated temperature). The remaining 10 incidents were product related, seven of which were technical: change in buffer consistency (*n* = 1), delayed migration (*n* = 2) (Fig 2), high invalid test rate (*n* = 1), poor background clearing (*n* = 2), and dust in cassette packaging (*n* = 1). The three remaining incidents concerned changes in IFU: two changes related to sample volume and one to reading time. None of the concerned IFU displayed track changes or a revision history, which would allow quick and accurate identification of the changes. Only one IFU displayed a new version number, and only two IFU showed a different date of issue (Fig 3). The changes had not been communicated to the user by writing or by an alert on the box.

For six of the incidents, we contacted the manufacturers (*n* = 3). For four incidents, the manufacturers’ reply was swift and correct: they provided information on changes in the IFU (*n* = 1) and on temperature stability (*n* = 1), and they performed corrective actions (*n* = 2, replacement of affected RDT kits, provision of additional buffer vials). For two incidents, the reply was inadequate. One reply was correct but delayed for 12 days (ascribed to summer holidays). For the second incident, the manufacturer replied that the validation of the new reading time should be done by us or that, alternatively, we could simply disregard the new IFU.

Overall, at least one quality problem was detected for each of the 11 RDTs. There was no clear overlap between different types of incidents: problems occurred with all RDT types and all manufacturers. To our knowledge, the observed shortcomings did not cause serious adverse events for participants (most of these RDTs were used as index tests and not to guide patient care), nor did they affect study data reliability.

## Discussion

This case study raises the issue of quality and postmarket surveillance of RDTs for neglected infectious diseases, irrespective of their diagnostic performance. When RDTs are put under scrutiny, shortcomings appear to be common. Some of the problems also occur with CE-marked RDTs. This is not surprising because in the current European Union regulation, IVDs for neglected infectious diseases are not considered to be high risk, and consequently, CE marks can be obtained by so-called self-certification by the manufacturer [10]. Of note, only small investments would be required from the manufacturers to correct the shortcomings here described.

The most worrying shortcomings are the changes in reading time and sample volume without notification to the user. Such changes are categorised as substantial because they may influence RDT performance [11]. One of these incidents illustrates the failure of the manufacturer to correctly handle a customer complaint: the attempt to shift responsibility for the validation of the changed reading time towards the user and the recommendation to disregard the IFU are unacceptable. At the users’ end, quality assurance systems should clearly include proactive monitoring of IFU changes. In line with this, the ISO 15189 guideline that has been developed for medical laboratories (end users of diagnostic devises) recommends “document control”—i.e., the prospective monitoring, updating, and archiving of all documents that may vary in versions or time, including IFU [4].

Limitations of this case study include the relatively small number of RDTs evaluated and the fact that not all elements of RDT quality were assessed. Likewise, one might debate the relevance of the findings, as the reported shortcomings did not cause any apparent serious adverse events. However, one should note that the possible effects of these shortcomings actually were mitigated by the study-related standard operating procedures in case of the shortcomings in labelling and IFU and by quick and appropriate action of the study team in case of RDT-related incidents. In support of its relevance, this study assessed real-life RDT products, and the product-related shortcomings reported here were observed thanks to the quality system of the clinical study. We expect that in the daily practice of a remote or low-resource setting, these shortcomings would not have been detected nor reported and may affect RDT performance and impact patient outcome.

Similar quality problems have been reported for RDTs targeting other infectious diseases. For example, in a study of 42 different malaria RDTs, shortcomings were found at the level of devices, buffers, packaging, labelling, and IFU [12]. For malaria and other frequent infections such as HIV and hepatitis B and C, WHO has taken a leading role in quality assurance of IVDs through prequalification and guidance on postmarket surveillance [6, 7, 11]. In the case of RDTs for neglected infectious diseases, such formal and international quality assurance mechanisms are less strong [13]. Moreover, stakeholders in this field may be less coordinated and less informed than those in the fields of malaria or HIV, for which national control programmes, funding agencies, nongovernmental organizations, and product experts constitute influential stakeholders. In the context of RDTs for neglected infectious diseases, production volumes are low, commercial interests are poor, and manufacturers may have limited ISO competence. At the other end of the supply chain, users of such RDTs tend to be dispersed and poorly trained [14]. At the same time, the requirements for these RDTs are particularly high: they should be user-friendly, withstand extreme environmental conditions, and be valid in heterogeneous patient populations [10].

Based on this experience, we recommend proactive interest from IVD manufacturers in line with ISO 13485 guidelines. Likewise, users even in remote settings should be motivated and trained to take up their role as emancipated customers. This can be achieved through biomedical curricula, national health authorities, and regional professional and scientific organisations such as the African Society for Laboratory Medicine. In addition, stakeholders involved in selection, procurement, and implementation of IVDs should be aware about these quality issues. Finally, regulatory authorities could subject RDTs for moderate-risk infectious diseases to a more rigorous premarketing scrutiny [13].

## Supporting Information

S1 Table Checklist for rapid diagnostic tests used as index tests in NIDIAG persistent fever study (XLSX)
